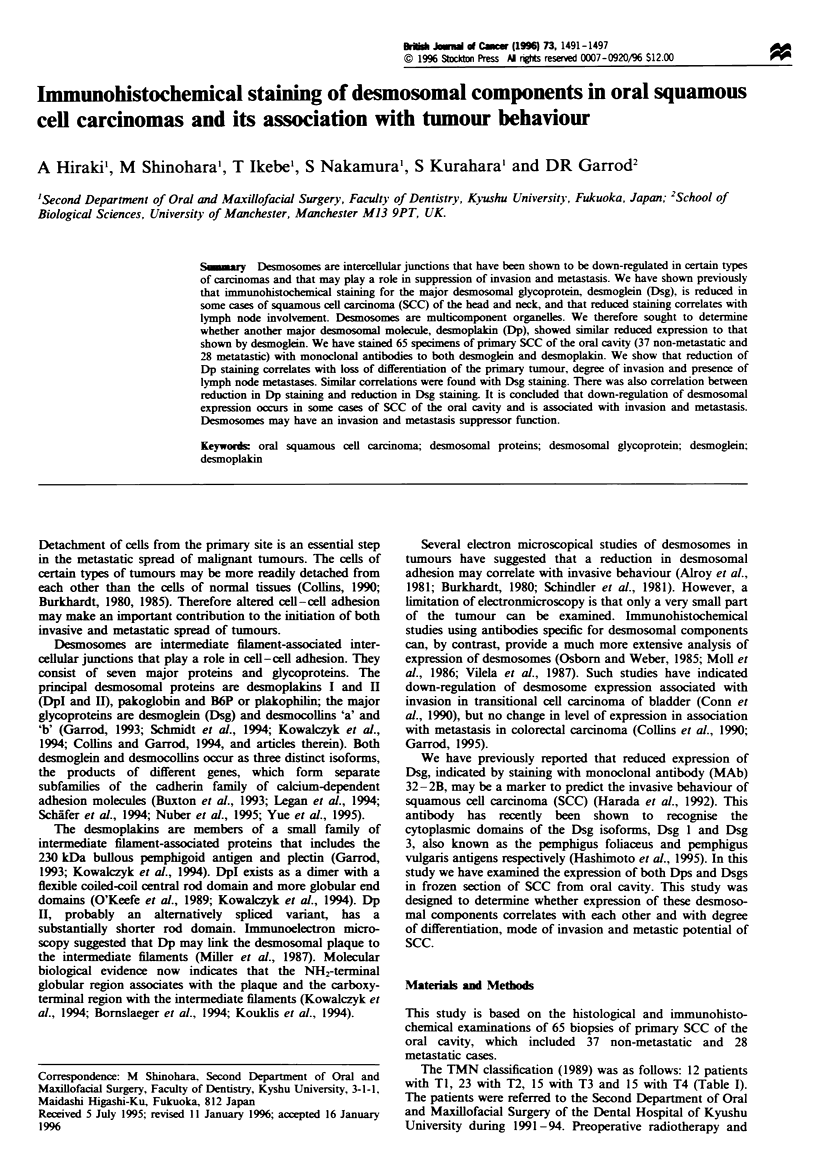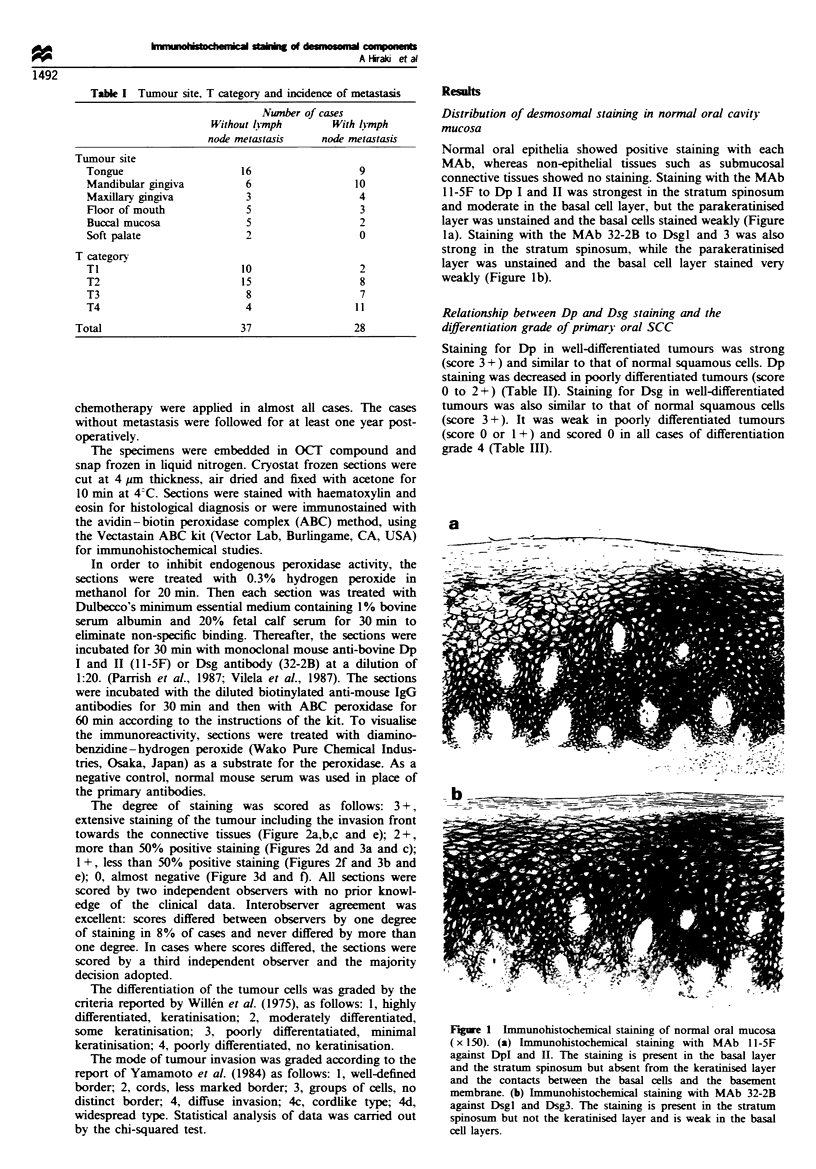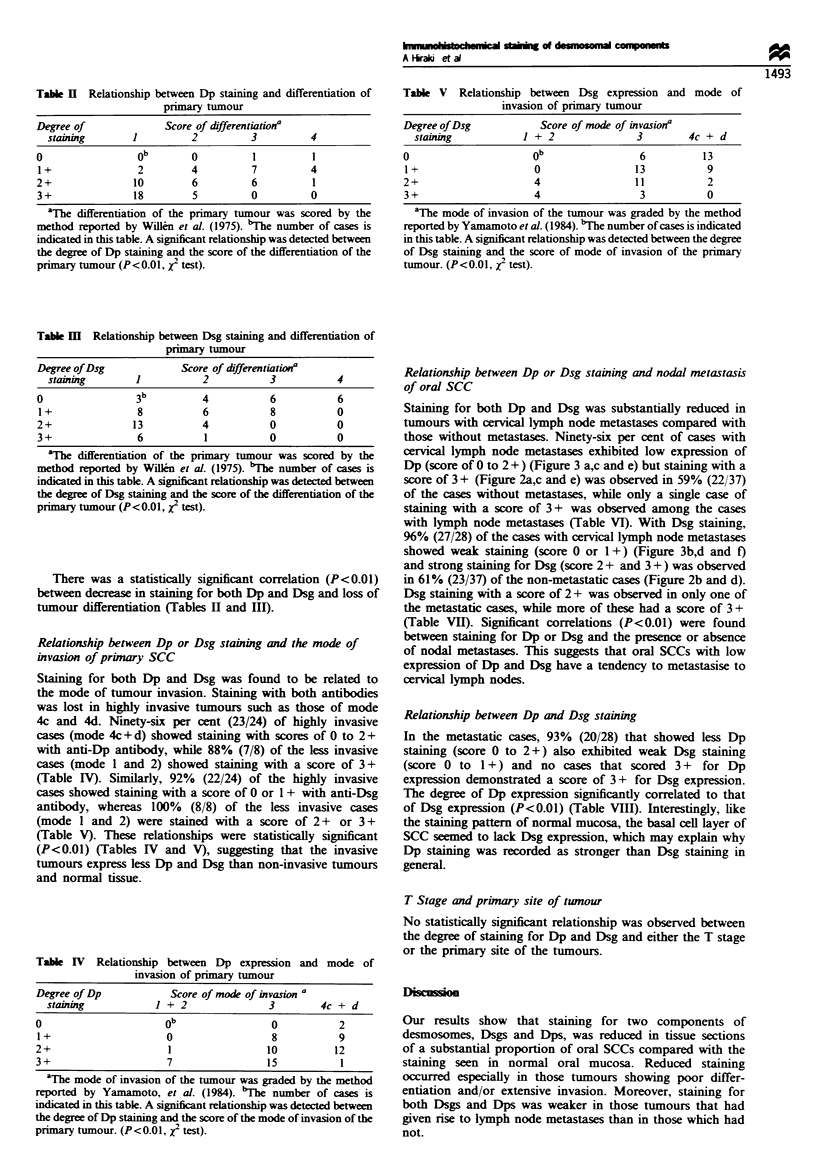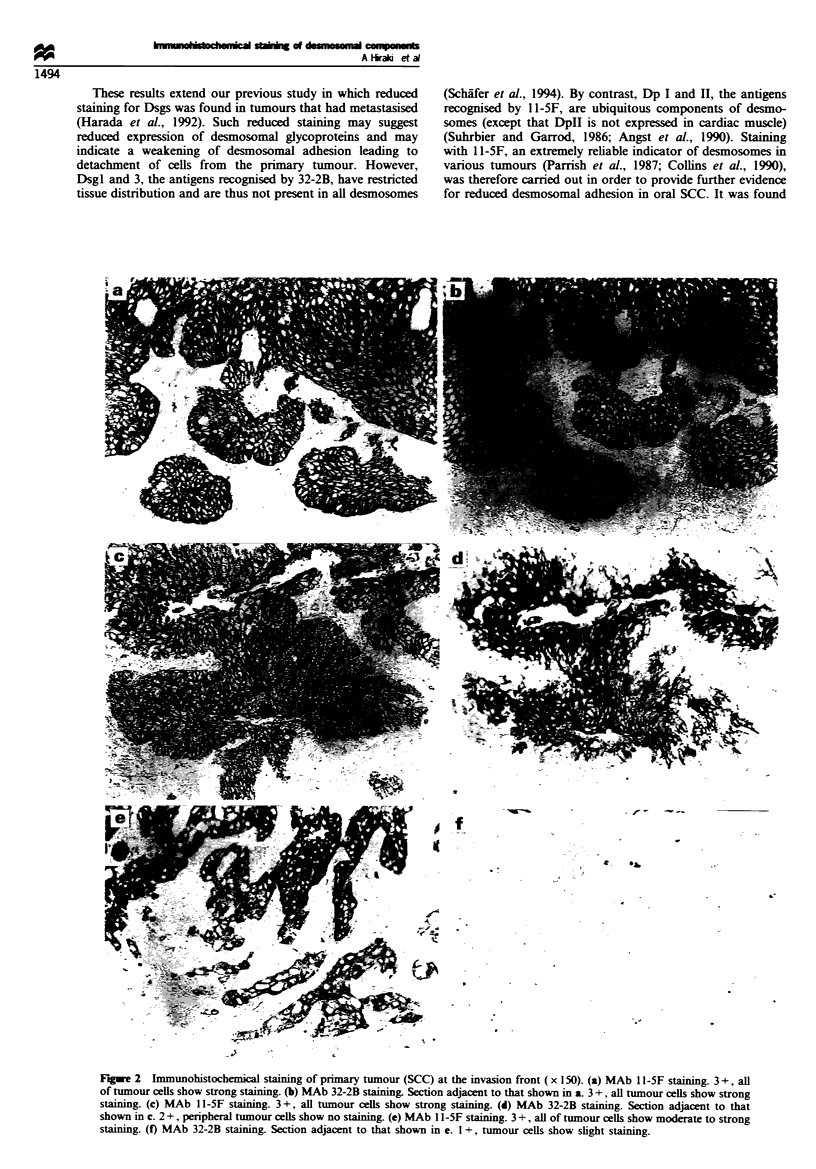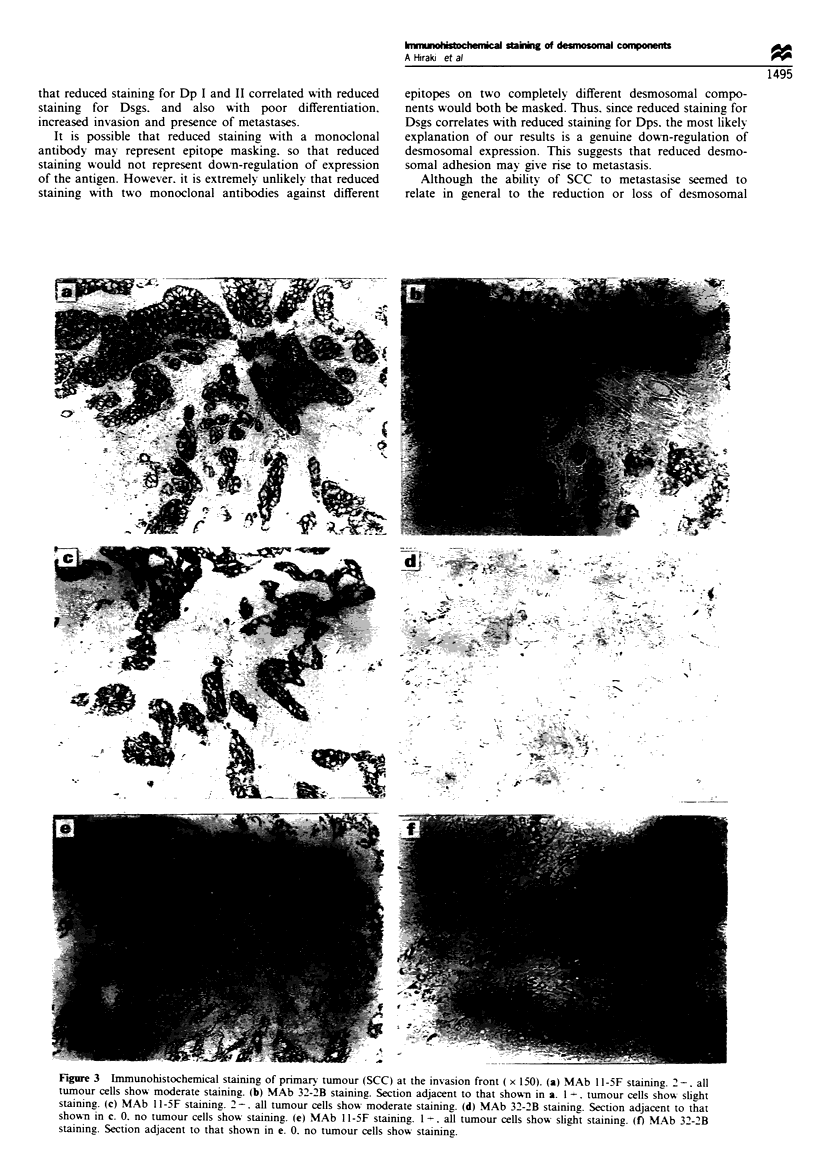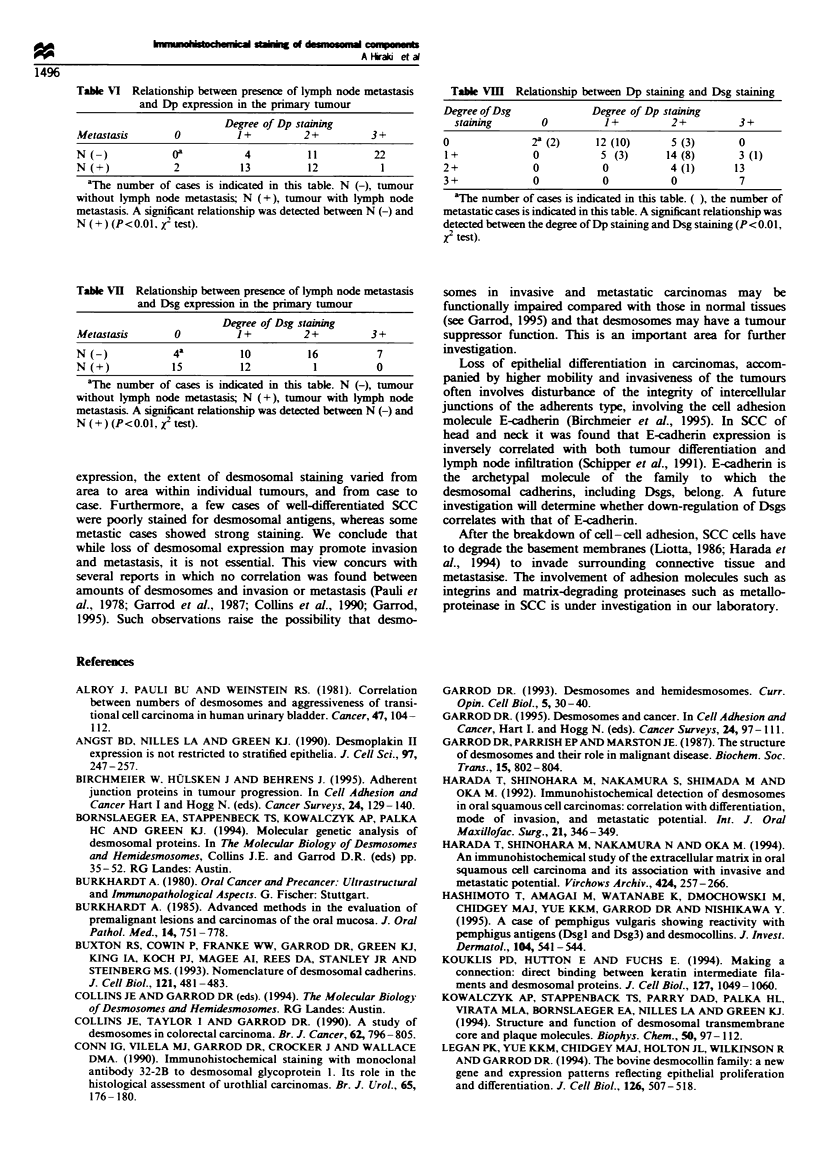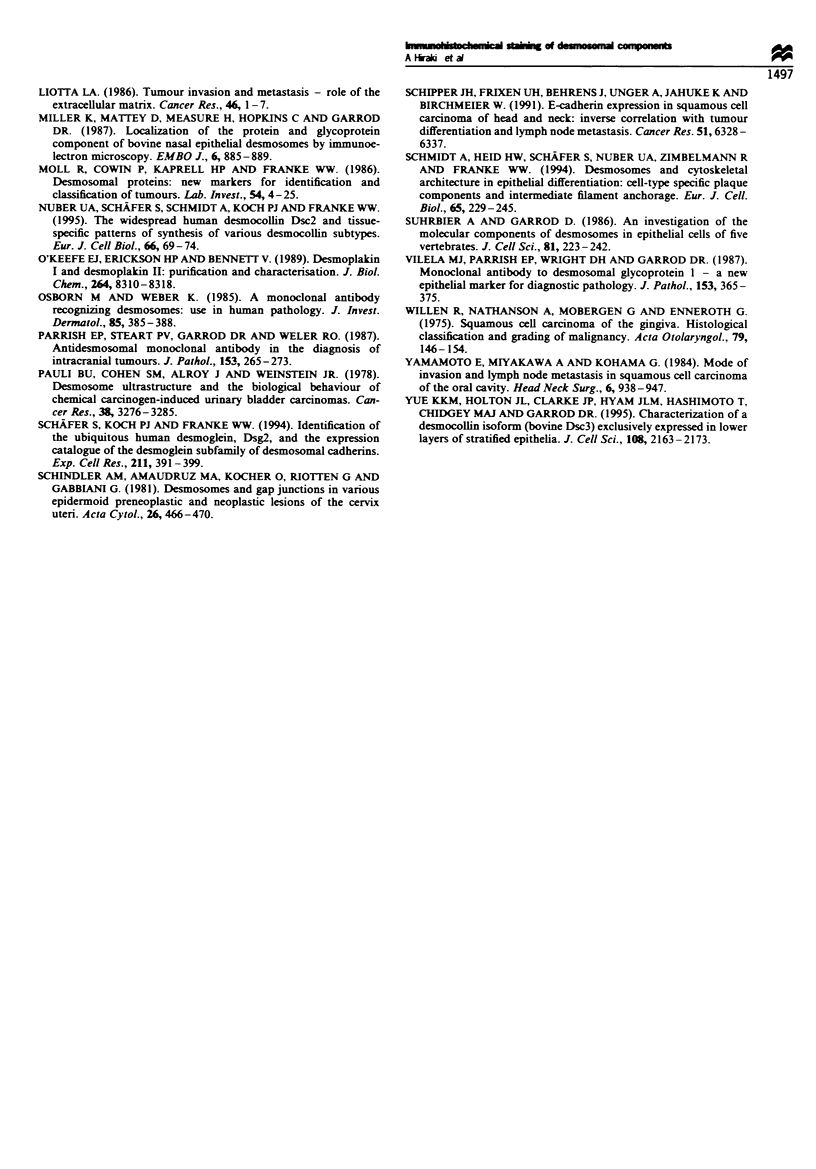# Immunohistochemical staining of desmosomal components in oral squamous cell carcinomas and its association with tumour behaviour.

**DOI:** 10.1038/bjc.1996.282

**Published:** 1996-06

**Authors:** A. Hiraki, M. Shinohara, T. Ikebe, S. Nakamura, S. Kurahara, D. R. Garrod

**Affiliations:** Second Department of Oral and Maxillofacial Surgery, Faculty of Dentistry, Kyushu University, Fukuoka, Japan.

## Abstract

**Images:**


British Journal of Cancer (1996) 73, 1491-1497

© 1996 Stockton Press All rights reserved 0007-0920/96 $12.00

### Immunohistochemical staining of desmosomal components in oral squamous cell carcinomas and its association with tumour behaviour

A Hiraki[1], M Shinohara[1], T Ikebe[1], S Nakamura[1], S Kurahara[1] and DR Garrod[2]

[1]*Second Department of Oral and Maxillofacial Surgery, Faculty of Dentistry, Kyushu University, Fukuoka, Japan;* [2]*School of Biological Sciences, University of Manchester, Manchester M13 9PT, UK.*

**Summary** Desmosomes are intercellular junctions that have been shown to be down-regulated in certain types of carcinomas and that may play a role in suppression of invasion and metastasis. We have shown previously that immunohistochemical staining for the major desmosomal glycoprotein, desmoglein (Dsg), is reduced in some cases of squamous cell carcinoma (SCC) of the head and neck, and that reduced staining correlates with lymph node involvement. Desmosomes are multicomponent organelles. We therefore sought to determine whether another major desmosomal molecule, desmoplakin (Dp), showed similar reduced expression to that shown by desmoglein. We have stained 65 specimens of primary SCC of the oral cavity (37 non-metastatic and 28 metatastic) with monoclonal antibodies to both desmoglein and desmoplakin. We show that reduction of Dp staining correlates with loss of differentiation of the primary tumour, degree of invasion and presence of lymph node metastases. Similar correlations were found with Dsg staining. There was also correlation between reduction in Dp staining and reduction in Dsg staining. It is concluded that down-regulation of desmosomal expression occurs in some cases of SCC of the oral cavity and is associated with invasion and metastasis. Desmosomes may have an invasion and metastasis suppressor function.

**Keywords:** oral squamous cell carcinoma; desmosomal proteins; desmosomal glycoprotein; desmoglein; desmoplakin

Detachment of cells from the primary site is an essential step in the metastatic spread of malignant tumours. The cells of certain types of tumours may be more readily detached from each other than the cells of normal tissues (Collins, 1990; Burkhardt, 1980, 1985). Therefore altered cell-cell adhesion may make an important contribution to the initiation of both invasive and metastatic spread of tumours.

Desmosomes are intermediate filament-associated intercellular junctions that play a role in cell-cell adhesion. They consist of seven major proteins and glycoproteins. The principal desmosomal proteins are desmoplakins I and II (DpI and II), pakoglobin and B6P or plakophilin; the major glycoproteins are desmoglein (Dsg) and desmocollins 'a' and 'b' (Garrod, 1993; Schmidt *et al.*, 1994; Kowalczyk *et al.*, 1994; Collins and Garrod, 1994, and articles therein). Both desmoglein and desmocollins occur as three distinct isoforms, the products of different genes, which form separate subfamilies of the cadherin family of calcium-dependent adhesion molecules (Buxton *et al.*, 1993; Legan *et al.*, 1994; Schäfer *et al.*, 1994; Nuber *et al.*, 1995; Yue *et al.*, 1995).

The desmoplakins are members of a small family of intermediate filament-associated proteins that includes the 230 kDa bullous pemphigoid antigen and plectin (Garrod, 1993; Kowalczyk *et al.*, 1994). DpI exists as a dimer with a flexible coiled-coil central rod domain and more globular end domains (O'Keefe *et al.*, 1989; Kowalczyk *et al.*, 1994). Dp II, probably an alternatively spliced variant, has a substantially shorter rod domain. Immunoelectron microscopy suggested that Dp may link the desmosomal plaque to the intermediate filaments (Miller *et al.*, 1987). Molecular biological evidence now indicates that the $NH_2$-terminal globular region associates with the plaque and the carboxy-terminal region with the intermediate filaments (Kowalczyk *et al.*, 1994; Bornslaeger *et al.*, 1994; Kouklis *et al.*, 1994).

Correspondence: M Shinohara, Second Department of Oral and Maxillofacial Surgery, Faculty of Dentistry, Kyshu University, 3-1-1, Maidashi Higashi-Ku, Fukuoka, 812 Japan
Received 5 July 1995; revised 11 January 1996; accepted 16 January 1996

Several electron microscopical studies of desmosomes in tumours have suggested that a reduction in desmosomal adhesion may correlate with invasive behaviour (Alroy *et al.*, 1981; Burkhardt, 1980; Schindler *et al.*, 1981). However, a limitation of electronmicroscopy is that only a very small part of the tumour can be examined. Immunohistochemical studies using antibodies specific for desmosomal components can, by contrast, provide a much more extensive analysis of expression of desmosomes (Osborn and Weber, 1985; Moll *et al.*, 1986; Vilela *et al.*, 1987). Such studies have indicated down-regulation of desmosome expression associated with invasion in transitional cell carcinoma of bladder (Conn *et al.*, 1990), but no change in level of expression in association with metastasis in colorectal carcinoma (Collins *et al.*, 1990; Garrod, 1995).

We have previously reported that reduced expression of Dsg, indicated by staining with monoclonal antibody (MAb) 32-2B, may be a marker to predict the invasive behaviour of squamous cell carcinoma (SCC) (Harada *et al.*, 1992). This antibody has recently been shown to recognise the cytoplasmic domains of the Dsg isoforms, Dsg 1 and Dsg 3, also known as the pemphigus foliaceus and pemphigus vulgaris antigens respectively (Hashimoto *et al.*, 1995). In this study we have examined the expression of both Dps and Dsgs in frozen section of SCC from oral cavity. This study was designed to determine whether expression of these desmosomal components correlates with each other and with degree of differentiation, mode of invasion and metastic potential of SCC.

### Materials and Methods

This study is based on the histological and immunohistochemical examinations of 65 biopsies of primary SCC of the oral cavity, which included 37 non-metastatic and 28 metastatic cases.

The TMN classification (1989) was as follows: 12 patients with T1, 23 with T2, 15 with T3 and 15 with T4 (Table I). The patients were referred to the Second Department of Oral and Maxillofacial Surgery of the Dental Hospital of Kyushu University during 1991-94. Preoperative radiotherapy and

**Table I** Tumour site, T category and incidence of metastasis

| | Number of cases | |
|---|---|---|
| | Without lymph node metastasis | With lymph node metastasis |
| Tumour site | | |
| Tongue | 16 | 9 |
| Mandibular gingiva | 6 | 10 |
| Maxillary gingiva | 3 | 4 |
| Floor of mouth | 5 | 3 |
| Buccal mucosa | 5 | 2 |
| Soft palate | 2 | 0 |
| T category | | |
| T1 | 10 | 2 |
| T2 | 15 | 8 |
| T3 | 8 | 7 |
| T4 | 4 | 11 |
| Total | 37 | 28 |

chemotherapy were applied in almost all cases. The cases without metastasis were followed for at least one year post-operatively.

The specimens were embedded in OCT compound and snap frozen in liquid nitrogen. Cryostat frozen sections were cut at 4 μm thickness, air dried and fixed with acetone for 10 min at 4°C. Sections were stained with haematoxylin and eosin for histological diagnosis or were immunostained with the avidin–biotin peroxidase complex (ABC) method, using the Vectastain ABC kit (Vector Lab, Burlingame, CA, USA) for immunohistochemical studies.

In order to inhibit endogenous peroxidase activity, the sections were treated with 0.3% hydrogen peroxide in methanol for 20 min. Then each section was treated with Dulbecco's minimum essential medium containing 1% bovine serum albumin and 20% fetal calf serum for 30 min to eliminate non-specific binding. Thereafter, the sections were incubated for 30 min with monoclonal mouse anti-bovine Dp I and II (11-5F) or Dsg antibody (32-2B) at a dilution of 1:20. (Parrish *et al*., 1987; Vilela *et al*., 1987). The sections were incubated with the diluted biotinylated anti-mouse IgG antibodies for 30 min and then with ABC peroxidase for 60 min according to the instructions of the kit. To visualise the immunoreactivity, sections were treated with diaminobenzidine–hydrogen peroxide (Wako Pure Chemical Industries, Osaka, Japan) as a substrate for the peroxidase. As a negative control, normal mouse serum was used in place of the primary antibodies.

The degree of staining was scored as follows: 3+, extensive staining of the tumour including the invasion front towards the connective tissues (Figure 2a,b,c and e); 2+, more than 50% positive staining (Figures 2d and 3a and c); 1+, less than 50% positive staining (Figures 2f and 3b and e); 0, almost negative (Figure 3d and f). All sections were scored by two independent observers with no prior knowledge of the clinical data. Interobserver agreement was excellent: scores differed between observers by one degree of staining in 8% of cases and never differed by more than one degree. In cases where scores differed, the sections were scored by a third independent observer and the majority decision adopted.

The differentiation of the tumour cells was graded by the criteria reported by Willén *et al*. (1975), as follows: 1, highly differentiated, keratinisation; 2, moderately differentiated, some keratinisation; 3, poorly differentatiated, minimal keratinisation; 4, poorly differentiated, no keratinisation.

The mode of tumour invasion was graded according to the report of Yamamoto *et al*. (1984) as follows: 1, well-defined border; 2, cords, less marked border; 3, groups of cells, no distinct border; 4, diffuse invasion; 4c, cordlike type; 4d, widespread type. Statistical analysis of data was carried out by the chi-squared test.

### Results

*Distribution of desmosomal staining in normal oral cavity mucosa*

Normal oral epithelia showed positive staining with each MAb, whereas non-epithelial tissues such as submucosal connective tissues showed no staining. Staining with the MAb 11-5F to Dp I and II was strongest in the stratum spinosum and moderate in the basal cell layer, but the parakeratinised layer was unstained and the basal cells stained weakly (Figure 1a). Staining with the MAb 32-2B to Dsg1 and 3 was also strong in the stratum spinosum, while the parakeratinised layer was unstained and the basal cell layer stained very weakly (Figure 1b).

*Relationship between Dp and Dsg staining and the differentiation grade of primary oral SCC*

Staining for Dp in well-differentiated tumours was strong (score 3+) and similar to that of normal squamous cells. Dp staining was decreased in poorly differentiated tumours (score 0 to 2+) (Table II). Staining for Dsg in well-differentiated tumours was also similar to that of normal squamous cells (score 3+). It was weak in poorly differentiated tumours (score 0 or 1+) and scored 0 in all cases of differentiation grade 4 (Table III).

**Figure 1** Immunohistochemical staining of normal oral mucosa (×150). (**a**) Immunohistochemical staining with MAb 11-5F against DpI and II. The staining is present in the basal layer and the stratum spinosum but absent from the keratinised layer and the contacts between the basal cells and the basement membrane. (**b**) Immunohistochemical staining with MAb 32-2B against Dsg1 and Dsg3. The staining is present in the stratum spinosum but not the keratinised layer and is weak in the basal cell layers.

**Table II** Relationship between Dp staining and differentiation of primary tumour

| Degree of staining | Score of differentiation[a] 1 | 2 | 3 | 4 |
|---|---|---|---|---|
| 0 | 0[b] | 0 | 1 | 1 |
| 1+ | 2 | 4 | 7 | 4 |
| 2+ | 10 | 6 | 6 | 1 |
| 3+ | 18 | 5 | 0 | 0 |

[a]The differentiation of the primary tumour was scored by the method reported by Willén *et al.* (1975). [b]The number of cases is indicated in this table. A significant relationship was detected between the degree of Dp staining and the score of the differentiation of the primary tumour ($P<0.01$, $\chi^2$ test).

**Table III** Relationship between Dsg staining and differentiation of primary tumour

| Degree of Dsg staining | Score of differentiation[a] 1 | 2 | 3 | 4 |
|---|---|---|---|---|
| 0 | 3[b] | 4 | 6 | 6 |
| 1+ | 8 | 6 | 8 | 0 |
| 2+ | 13 | 4 | 0 | 0 |
| 3+ | 6 | 1 | 0 | 0 |

[a]The differentiation of the primary tumour was scored by the method reported by Willén *et al.* (1975). [b]The number of cases is indicated in this table. A significant relationship was detected between the degree of Dsg staining and the score of the differentiation of the primary tumour ($P<0.01$, $\chi^2$ test).

There was a statistically significant correlation ($P<0.01$) between decrease in staining for both Dp and Dsg and loss of tumour differentiation (Tables II and III).

*Relationship between Dp or Dsg staining and the mode of invasion of primary SCC*

Staining for both Dp and Dsg was found to be related to the mode of tumour invasion. Staining with both antibodies was lost in highly invasive tumours such as those of mode 4c and 4d. Ninety-six per cent (23/24) of highly invasive cases (mode 4c+d) showed staining with scores of 0 to 2+ with anti-Dp antibody, while 88% (7/8) of the less invasive cases (mode 1 and 2) showed staining with a score of 3+ (Table IV). Similarly, 92% (22/24) of the highly invasive cases showed staining with a score of 0 or 1+ with anti-Dsg antibody, whereas 100% (8/8) of the less invasive cases (mode 1 and 2) were stained with a score of 2+ or 3+ (Table V). These relationships were statistically significant ($P<0.01$) (Tables IV and V), suggesting that the invasive tumours express less Dp and Dsg than non-invasive tumours and normal tissue.

**Table IV** Relationship between Dp expression and mode of invasion of primary tumour

| Degree of Dp staining | Score of mode of invasion[a] 1 + 2 | 3 | 4c + d |
|---|---|---|---|
| 0 | 0[b] | 0 | 2 |
| 1+ | 0 | 8 | 9 |
| 2+ | 1 | 10 | 12 |
| 3+ | 7 | 15 | 1 |

[a]The mode of invasion of the tumour was graded by the method reported by Yamamoto, *et al.* (1984). [b]The number of cases is indicated in this table. A significant relationship was detected between the degree of Dp staining and the score of the mode of invasion of the primary tumour. ($P<0.01$, $\chi^2$ test).

**Table V** Relationship between Dsg expression and mode of invasion of primary tumour

| Degree of Dsg staining | Score of mode of invasion[a] 1 + 2 | 3 | 4c + d |
|---|---|---|---|
| 0 | 0[b] | 6 | 13 |
| 1+ | 0 | 13 | 9 |
| 2+ | 4 | 11 | 2 |
| 3+ | 4 | 3 | 0 |

[a]The mode of invasion of the tumour was graded by the method reported by Yamamoto *et al.* (1984). [b]The number of cases is indicated in this table. A significant relationship was detected between the degree of Dsg staining and the score of mode of invasion of the primary tumour. ($P<0.01$, $\chi^2$ test).

*Relationship between Dp or Dsg staining and nodal metastasis of oral SCC*

Staining for both Dp and Dsg was substantially reduced in tumours with cervical lymph node metastases compared with those without metastases. Ninety-six per cent of cases with cervical lymph node metastases exhibited low expression of Dp (score of 0 to 2+) (Figure 3 a,c and e) but staining with a score of 3+ (Figure 2a,c and e) was observed in 59% (22/37) of the cases without metastases, while only a single case of staining with a score of 3+ was observed among the cases with lymph node metastases (Table VI). With Dsg staining, 96% (27/28) of the cases with cervical lymph node metastases showed weak staining (score 0 or 1+) (Figure 3b,d and f) and strong staining for Dsg (score 2+ and 3+) was observed in 61% (23/37) of the non-metastatic cases (Figure 2b and d). Dsg staining with a score of 2+ was observed in only one of the metastatic cases, while more of these had a score of 3+ (Table VII). Significant correlations ($P<0.01$) were found between staining for Dp or Dsg and the presence or absence of nodal metastases. This suggests that oral SCCs with low expression of Dp and Dsg have a tendency to metastasise to cervical lymph nodes.

*Relationship between Dp and Dsg staining*

In the metastatic cases, 93% (20/28) that showed less Dp staining (score 0 to 2+) also exhibited weak Dsg staining (score 0 to 1+) and no cases that scored 3+ for Dp expression demonstrated a score of 3+ for Dsg expression. The degree of Dp expression significantly correlated to that of Dsg expression ($P<0.01$) (Table VIII). Interestingly, like the staining pattern of normal mucosa, the basal cell layer of SCC seemed to lack Dsg expression, which may explain why Dp staining was recorded as stronger than Dsg staining in general.

*T Stage and primary site of tumour*

No statistically significant relationship was observed between the degree of staining for Dp and Dsg and either the T stage or the primary site of the tumours.

### Discussion

Our results show that staining for two components of desmosomes, Dsgs and Dps, was reduced in tissue sections of a substantial proportion of oral SCCs compared with the staining seen in normal oral mucosa. Reduced staining occurred especially in those tumours showing poor differentiation and/or extensive invasion. Moreover, staining for both Dsgs and Dps was weaker in those tumours that had given rise to lymph node metastases than in those which had not.

These results extend our previous study in which reduced staining for Dsgs was found in tumours that had metastasised (Harada *et al.*, 1992). Such reduced staining may suggest reduced expression of desmosomal glycoproteins and may indicate a weakening of desmosomal adhesion leading to detachment of cells from the primary tumour. However, Dsg1 and 3, the antigens recognised by 32-2B, have restricted tissue distribution and are thus not present in all desmosomes (Schäfer *et al.*, 1994). By contrast, Dp I and II, the antigens recognised by 11-5F, are ubiquitous components of desmosomes (except that DpII is not expressed in cardiac muscle) (Suhrbier and Garrod, 1986; Angst *et al.*, 1990). Staining with 11-5F, an extremely reliable indicator of desmosomes in various tumours (Parrish *et al.*, 1987; Collins *et al.*, 1990), was therefore carried out in order to provide further evidence for reduced desmosomal adhesion in oral SCC. It was found

**Figure 2** Immunohistochemical staining of primary tumour (SCC) at the invasion front (×150). (**a**) MAb 11-5F staining. 3+, all of tumour cells show strong staining. (**b**) MAb 32-2B staining. Section adjacent to that shown in **a**. 3+, all tumour cells show strong staining. (**c**) MAb 11-5F staining. 3+, all tumour cells show strong staining. (**d**) MAb 32-2B staining. Section adjacent to that shown in **c**. 2+, peripheral tumour cells show no staining. (**e**) MAb 11-5F staining. 3+, all of tumour cells show moderate to strong staining. (**f**) MAb 32-2B staining. Section adjacent to that shown in **e**. 1+, tumour cells show slight staining.

that reduced staining for Dp I and II correlated with reduced staining for Dsgs, and also with poor differentiation, increased invasion and presence of metastases.

It is possible that reduced staining with a monoclonal antibody may represent epitope masking, so that reduced staining would not represent down-regulation of expression of the antigen. However, it is extremely unlikely that reduced staining with two monoclonal antibodies against different epitopes on two completely different desmosomal components would both be masked. Thus, since reduced staining for Dsgs correlates with reduced staining for Dps, the most likely explanation of our results is a genuine down-regulation of desmosomal expression. This suggests that reduced desmosomal adhesion may give rise to metastasis.

Although the ability of SCC to metastasise seemed to relate in general to the reduction or loss of desmosomal

**Figure 3** Immunohistochemical staining of primary tumour (SCC) at the invasion front ( × 150). (**a**) MAb 11-5F staining. 2 – , all tumour cells show moderate staining. (**b**) MAb 32-2B staining. Section adjacent to that shown in **a**. 1 + , tumour cells show slight staining. (**c**) MAb 11-5F staining. 2 – , all tumour cells show moderate staining. (**d**) MAb 32-2B staining. Section adjacent to that shown in **c**. 0, no tumour cells show staining. (**e**) MAb 11-5F staining. 1 + , all tumour cells show slight staining. (**f**) MAb 32-2B staining. Section adjacent to that shown in **e**. 0, no tumour cells show staining.

Table VI Relationship between presence of lymph node metastasis and Dp expression in the primary tumour

| Metastasis | Degree of Dp staining 0 | 1+ | 2+ | 3+ |
|---|---|---|---|---|
| N (−) | 0[a] | 4 | 11 | 22 |
| N (+) | 2 | 13 | 12 | 1 |

[a]The number of cases is indicated in this table. N (−), tumour without lymph node metastasis; N (+), tumour with lymph node metastasis. A significant relationship was detected between N (−) and N (+) ($P<0.01$, $\chi^2$ test).

Table VIII Relationship between Dp staining and Dsg staining

| Degree of Dsg staining | Degree of Dp staining 0 | 1+ | 2+ | 3+ |
|---|---|---|---|---|
| 0 | 2[a] (2) | 12 (10) | 5 (3) | 0 |
| 1+ | 0 | 5 (3) | 14 (8) | 3 (1) |
| 2+ | 0 | 0 | 4 (1) | 13 |
| 3+ | 0 | 0 | 0 | 7 |

[a]The number of cases is indicated in this table. ( ), the number of metastatic cases is indicated in this table. A significant relationship was detected between the degree of Dp staining and Dsg staining ($P<0.01$, $\chi^2$ test).

Table VII Relationship between presence of lymph node metastasis and Dsg expression in the primary tumour

| Metastasis | Degree of Dsg staining 0 | 1+ | 2+ | 3+ |
|---|---|---|---|---|
| N (−) | 4[a] | 10 | 16 | 7 |
| N (+) | 15 | 12 | 1 | 0 |

[a]The number of cases is indicated in this table. N (−), tumour without lymph node metastasis; N (+), tumour with lymph node metastasis. A significant relationship was detected between N (−) and N (+) ($P<0.01$, $\chi^2$ test).

expression, the extent of desmosomal staining varied from area to area within individual tumours, and from case to case. Furthermore, a few cases of well-differentiated SCC were poorly stained for desmosomal antigens, whereas some metastic cases showed strong staining. We conclude that while loss of desmosomal expression may promote invasion and metastasis, it is not essential. This view concurs with several reports in which no correlation was found between amounts of desmosomes and invasion or metastasis (Pauli *et al*., 1978; Garrod *et al*., 1987; Collins *et al*., 1990; Garrod, 1995). Such observations raise the possibility that desmosomes in invasive and metastatic carcinomas may be functionally impaired compared with those in normal tissues (see Garrod, 1995) and that desmosomes may have a tumour suppressor function. This is an important area for further investigation.

Loss of epithelial differentiation in carcinomas, accompanied by higher mobility and invasiveness of the tumours often involves disturbance of the integrity of intercellular junctions of the adherents type, involving the cell adhesion molecule E-cadherin (Birchmeier *et al*., 1995). In SCC of head and neck it was found that E-cadherin expression is inversely correlated with both tumour differentiation and lymph node infiltration (Schipper *et al*., 1991). E-cadherin is the archetypal molecule of the family to which the desmosomal cadherins, including Dsgs, belong. A future investigation will determine whether down-regulation of Dsgs correlates with that of E-cadherin.

After the breakdown of cell–cell adhesion, SCC cells have to degrade the basement membranes (Liotta, 1986; Harada *et al*., 1994) to invade surrounding connective tissue and metastasise. The involvement of adhesion molecules such as integrins and matrix-degrading proteinases such as metalloproteinase in SCC is under investigation in our laboratory.

### References

ALROY J, PAULI BU AND WEINSTEIN RS. (1981). Correlation between numbers of desmosomes and aggressiveness of transitional cell carcinoma in human urinary bladder. *Cancer*, **47**, 104–112.

ANGST BD, NILLES LA AND GREEN KJ. (1990). Desmoplakin II expression is not restricted to stratified epithelia. *J. Cell Sci*., **97**, 247–257.

BIRCHMEIER W, HÜLSKEN J AND BEHRENS J. (1995). Adherent junction proteins in tumour progression. In *Cell Adhesion and Cancer* Hart I and Hogg N. (eds). *Cancer Surveys*, **24**, 129–140.

BORNSLAEGER EA, STAPPENBECK TS, KOWALCZYK AP, PALKA HC AND GREEN KJ. (1994). Molecular genetic analysis of desmosomal proteins. In *The Molecular Biology of Desmosomes and Hemidesmosomes*, Collins J.E. and Garrod D.R. (eds) pp. 35–52. RG Landes: Austin.

BURKHARDT A. (1980). *Oral Cancer and Precancer: Ultrastructural* and *Immunopathological Aspects*. G. Fischer: Stuttgart.

BURKHARDT A. (1985). Advanced methods in the evaluation of premalignant lesions and carcinomas of the oral mucosa. *J. Oral Pathol. Med*., **14**, 751–778.

BUXTON RS, COWIN P, FRANKE WW, GARROD DR, GREEN KJ, KING IA, KOCH PJ, MAGEE AI, REES DA, STANLEY JR AND STEINBERG MS. (1993). Nomenclature of desmosomal cadherins. *J. Cell Biol*., **121**, 481–483.

COLLINS JE AND GARROD DR (eds). (1994). *The Molecular Biology of Desmosomes and Hemidesmosomes*. RG Landes: Austin.

COLLINS JE, TAYLOR I AND GARROD DR. (1990). A study of desmosomes in colorectal carcinoma. *Br. J. Cancer*, **62**, 796–805.

CONN IG, VILELA MJ, GARROD DR, CROCKER J AND WALLACE DMA. (1990). Immunohistochemical staining with monoclonal antibody 32-2B to desmosomal glycoprotein 1. Its role in the histological assessment of urothlial carcinomas. *Br. J. Urol*., **65**, 176–180.

GARROD DR. (1993). Desmosomes and hemidesmosomes. *Curr. Opin. Cell Biol*., **5**, 30–40.

GARROD DR. (1995). Desmosomes and cancer. In *Cell Adhesion and Cancer*, Hart I. and Hogg N. (eds). *Cancer Surveys*, **24**, 97–111.

GARROD DR, PARRISH EP AND MARSTON JE. (1987). The structure of desmosomes and their role in malignant disease. *Biochem. Soc. Trans*., **15**, 802–804.

HARADA T, SHINOHARA M, NAKAMURA S, SHIMADA M AND OKA M. (1992). Immunohistochemical detection of desmosomes in oral squamous cell carcinomas: correlation with differentiation, mode of invasion, and metastatic potential. *Int. J. Oral Maxillofac. Surg*., **21**, 346–349.

HARADA T, SHINOHARA M, NAKAMURA N AND OKA M. (1994). An immunohistochemical study of the extracellular matrix in oral squamous cell carcinoma and its association with invasive and metastatic potential. *Virchows Archiv*., **424**, 257–266.

HASHIMOTO T, AMAGAI M, WATANABE K, DMOCHOWSKI M, CHIDGEY MAJ, YUE KKM, GARROD DR AND NISHIKAWA Y. (1995). A case of pemphigus vulgaris showing reactivity with pemphigus antigens (Dsg1 and Dsg3) and desmocollins. *J. Invest. Dermatol*., **104**, 541–544.

KOUKLIS PD, HUTTON E AND FUCHS E. (1994). Making a connection: direct binding between keratin intermediate filaments and desmosomal proteins. *J. Cell Biol*., **127**, 1049–1060.

KOWALCZYK AP, STAPPENBACK TS, PARRY DAD, PALKA HL, VIRATA MLA, BORNSLAEGER EA, NILLES LA AND GREEN KJ. (1994). Structure and function of desmosomal transmembrane core and plaque molecules. *Biophys. Chem*., **50**, 97–112.

LEGAN PK, YUE KKM, CHIDGEY MAJ, HOLTON JL, WILKINSON R AND GARROD DR. (1994). The bovine desmocollin family: a new gene and expression patterns reflecting epithelial proliferation and differentiation. *J. Cell Biol*., **126**, 507–518.

LIOTTA LA. (1986). Tumour invasion and metastasis – role of the extracellular matrix. *Cancer Res.*, **46**, 1-7.
MILLER K, MATTEY D, MEASURE H, HOPKINS C AND GARROD DR. (1987). Localization of the protein and glycoprotein component of bovine nasal epithelial desmosomes by immunoelectron microscopy. *EMBO J.*, **6**, 885-889.
MOLL R, COWIN P, KAPRELL HP AND FRANKE WW. (1986). Desmosomal proteins: new markers for identification and classification of tumours. *Lab. Invest.*, **54**, 4-25.
NUBER UA, SCHÄFER S, SCHMIDT A, KOCH PJ AND FRANKE WW. (1995). The widespread human desmocollin Dsc2 and tissue-specific patterns of synthesis of various desmocollin subtypes. *Eur. J. Cell Biol.*, **66**, 69-74.
O'KEEFE EJ, ERICKSON HP AND BENNETT V. (1989). Desmoplakin I and desmoplakin II: purification and characterisation. *J. Biol. Chem.*, **264**, 8310-8318.
OSBORN M AND WEBER K. (1985). A monoclonal antibody recognizing desmosomes: use in human pathology. *J. Invest. Dermatol.*, **85**, 385-388.
PARRISH EP, STEART PV, GARROD DR AND WELER RO. (1987). Antidesmosomal monoclonal antibody in the diagnosis of intracranial tumours. *J. Pathol.*, **153**, 265-273.
PAULI BU, COHEN SM, ALROY J AND WEINSTEIN JR. (1978). Desmosome ultrastructure and the biological behaviour of chemical carcinogen-induced urinary bladder carcinomas. *Cancer Res.*, **38**, 3276-3285.
SCHÄFER S, KOCH PJ AND FRANKE WW. (1994). Identification of the ubiquitous human desmoglein, Dsg2, and the expression catalogue of the desmoglein subfamily of desmosomal cadherins. *Exp. Cell Res.*, **211**, 391-399.
SCHINDLER AM, AMAUDRUZ MA, KOCHER O, RIOTTEN G AND GABBIANI G. (1981). Desmosomes and gap junctions in various epidermoid preneoplastic and neoplastic lesions of the cervix uteri. *Acta Cytol.*, **26**, 466-470.
SCHIPPER JH, FRIXEN UH, BEHRENS J, UNGER A, JAHUKE K AND BIRCHMEIER W. (1991). E-cadherin expression in squamous cell carcinoma of head and neck: inverse correlation with tumour differentiation and lymph node metastasis. *Cancer Res.* **51**, 6328-6337.
SCHMIDT A, HEID HW, SCHÄFER S, NUBER UA, ZIMBELMANN R AND FRANKE WW. (1994). Desmosomes and cytoskeletal architecture in epithelial differentiation: cell-type specific plaque components and intermediate filament anchorage. *Eur. J. Cell. Biol.*, **65**, 229-245.
SUHRBIER A AND GARROD D. (1986). An investigation of the molecular components of desmosomes in epithelial cells of five vertebrates. *J. Cell Sci.*, **81**, 223-242.
VILELA MJ, PARRISH EP, WRIGHT DH AND GARROD DR. (1987). Monoclonal antibody to desmosomal glycoprotein 1 – a new epithelial marker for diagnostic pathology. *J. Pathol.*, **153**, 365-375.
WILLEN R, NATHANSON A, MOBERGEN G AND ENNEROTH G. (1975). Squamous cell carcinoma of the gingiva. Histological classification and grading of malignancy. *Acta Otolaryngol.*, **79**, 146-154.
YAMAMOTO E, MIYAKAWA A AND KOHAMA G. (1984). Mode of invasion and lymph node metastasis in squamous cell carcinoma of the oral cavity. *Head Neck Surg.*, **6**, 938-947.
YUE KKM, HOLTON JL, CLARKE JP, HYAM JLM, HASHIMOTO T, CHIDGEY MAJ AND GARROD DR. (1995). Characterization of a desmocollin isoform (bovine Dsc3) exclusively expressed in lower layers of stratified epithelia. *J. Cell Sci.*, **108**, 2163-2173.